# Supplementary material for: Epigenome-wide meta-analysis of PTSD across 10 military and civilian cohorts identifies methylation changes in AHRR
Source: Nat Commun. 2020 Nov 24;11:5965. doi: 10.1038/s41467-020-19615-x (PMC7686485; doi:10.1038/s41467-020-19615-x)
Supplement: Supplementary file 1 — Supplementary Information [file 41467_2020_19615_MOESM1_ESM.pdf]

Epigenome-wide meta-analysis of PTSD across 10 military and civilian cohorts identifies methylation differences in *AHRR*

Smith et al.

Supplementary Information

Supplementary Methods

Cohort descriptions

Supplementary Figures

Supplementary Figure 1. Forest Plots of association between PTSD and *AHRR* CpGs in each cohort.

Supplementary Figure 2. Forest Plots of association between PTSD and *AHRR* CpGs stratified by sex.

Supplementary Figure 3. Forest Plots of association between PTSD and *AHRR* CpGs stratified by European and African ancestry.

Supplementary Figure 4. Comparison of effect sizes before and after controlling for smoking.

Supplementary Figure 5. Forest Plots of association between PTSD and *AHRR* CpGs stratified by smokers vs nonsmokers.

Supplementary Figure 6. Association of Smoking-Associated CpG sites with current PTSD in analyses stratified by self-reported smoking status.

Supplementary Figure 7. Lower Kynurenine levels among PTSD cases in the MRS cohort.

Supplementary Figure 8. Correlation of cotinine and kynurenine across all MRS subjects.

Supplementary References

Supplementary Methods

Detroit Neighborhood Health Study (DNHS) - As detailed in a previous study [1], participants (n=1,547 at the baseline wave) were assessed for PTSD symptoms using the PTSD checklist (PCL-C), a 17-item self-report measure of Diagnostic and Statistical Manual of Mental Disorders (DSM-IV) symptoms, and additional questions about duration, timing, and impairment or disability due to the symptoms [2]. Participants were initially asked to identify Potentially Traumatic Events that they experienced in the past from a list of 19 events. PTSD symptoms were then assessed by referencing two traumatic events that the respondent may have experienced: one that the participant regarded as the worst and one randomly selected event from the remaining PTEs a respondent may have experienced. Respondents were considered affected by lifetime PTSD if all six DSM-IV criteria were met in reference to either the worst or the random event. The DNHS was approved by the institutional review board at the University of Michigan and University of North Carolina at Chapel Hill.

*Grady Trauma Project (GTP) –*GTP research participants were approached in the waiting rooms of the primary care clinic or obstetrical-gynecological clinic of a large, urban, public hospital in Atlanta, GA while either waiting for their medical appointments or while waiting with others who were scheduled for medical appointments [3]. Subjects willing to participate provided written informed consent and participated in a verbal interview and blood draw. Current and lifetime PTSD diagnosis was assessed by clinical psychologists using the Clinician-Administered PTSD Scale for DSM IV (CAPS-4) [4] or the Mini International Neuropsychiatric Interview DSM IV (MINI), an instrument designed to assess major Axis 1 disorders with high validity and reliability [5, 6]. For this study, cases were specified as having current PTSD, and controls had no current or lifetime history of the disorder. Demographic variables including age, sex and race were assessed through self-report. The Institutional Review Boards of Emory University School of Medicine and the Research Oversight Committee of Grady Memorial Hospital approved this study.

World Trade Center Responders (WTC) - As described by Bromet and colleagues [7, 8], responders enrolled in the Stony Brook University/Long Island WTC Health Program were administered the SCID PTSD module modified to assess PTSD in relation to WTC exposures. The sample providing blood samples for the epigenetics assays was restricted to men (the vast majority of the responders) and oversampled for posttraumatic stress disorder (PTSD) [9]. The Committees on Research Involving Human Subjects at Stony Brook University approved the study.

*Army Study to Assess Risk and Resilience in Servicemembers (Army STARRS):*Details of the diagnostic procedures are described in detail in prior STARRS publication [10]. Briefly, participants completed a computerized version of the Composite International Diagnostic Interview screening scales (CIDI-SC) and a screening version of the PTSD Checklist (PCL) for DSM-IV. Trauma exposure was assessed from answers pertaining to childhood, adulthood civilian, and military traumatic events. PTSD diagnosis was assigned using multiple imputation methods that relied on PCL and CIDI-SC data; our clinical reappraisal study found satisfactory concordance with independent clinical diagnoses based on blinded Structured Clinical Interviews for DSM-IV (AUC = 0.70–0.79; κ =0.4–0.6). Healthy controls were determined not to have PTSD using the same instruments and approach. The recruitment, consent, human subject and

data protection procedures were approved by the Human Subjects Committees of the Uniformed Services University of the Health Sciences for the Henry M. Jackson Foundation (the primary grantee), the Institute for Social Research at the University of Michigan (the organization collecting the data), and all other collaborating organizations.

Injury and Traumatic Stress (INTRuST): Patients and healthy controls were recruited from INTRuST clinical trials (with DNA collected prior to treatment) or specifically to contribute to the INTRuST biorepository. Patients were diagnosed with PTSD using study-specific measures which could have included either the CAPS-IV or CAPS-5, the MINI, or self-report using the PCL. Healthy controls were determined to be free of major psychiatric diagnoses, including PTSD, using the MINI or a comparable clinical interview. The Human Research Protection Program (HRPP) at UCSD approved this study, as did all the IRBs at participating sites.

Marine Resiliency Study (MRS) - In the MRS [11, 12], PTSD was diagnosed up to 3 times, once before deployment and 3 and/or 6 month post deployment. Post-traumatic stress (PTS) symptoms were assessed using a structured diagnostic interview, the Clinician Administered PTSD Scale (CAPS), and PTSD diagnosis followed the DSM-IV criteria for partial and full PTSD and a minimum CAPS score of 40. None of the participants in the EWAS and metabolome study had PTSD at pre-deployment. Samples (DNA from whole-blood and Li-heparin plasma for metabolome) of PTSD cases were selected from the 3- or 6-months post-deployment visits, choosing the visit with the highes CAPS score. Combat-exposed controls with low to no PTSD-symptoms were selected from matching post-deployment visits. The study was approved by the University of California – San Diego Institutional Review Board.

Prospective Research in Stress-related Military Operations (PRISMO) - All subjects in the DD were male participants in PRISMO, a large prospective study of 1,032 well-characterized Dutch military soldiers scheduled for a deployment of at least four months to Afghanistan with longitudinal follow-up. Baseline measures were recorded at one month before deployment. Follow-up was performed at one month and six months post-deployment, and data from the baseline and six-month follow-up were used for this analysis. A subset (total n=93) of three similarly sized subgroups of PRISMO study participants were pre-selected based on the level of traumatic stress exposure and the presence of PTSD symptoms: i) a subgroup showing high combat-trauma exposure (7.3 ± 2.9) and high levels of post-deployment PTSD symptoms (45.3 ± 8.6); ii) a subgroup showing high combat-trauma exposure (8.6 ± 2.3) and a low severity of PTSD symptoms (26.0 ± 3.7); and iii) a subgroup showing low combat-trauma exposure (0.4 ± 0.5) and low levels of post-deployment PTSD symptoms (25.1 ± 3.7).

Blood samples were collected six months after deployment. The blood cell-type composition was investigated using flow cytometry, implemented in the clinical laboratory of Utrecht University Medical Center, as previously reported [13]. The presence and severity of symptoms of PTSD over the previous four weeks were assessed with the 22-item Self-Report Inventory for PTSD (SRIP), which has good reliability and validity. Differences in PTSD symptoms between time points were log-transformed to improve the distribution. Exposure to traumatic stress during deployment was assessed with a 19-item deployment experiences checklist, as previously reported [14]. The study was approved by the Institutional Review Board of the University Medical Center Utrecht (Utrecht, the Netherlands). Written consent was also obtained.

Mid-Atlantic Mental Illness Research Education and Clinical Center PTSD Study (VA-M-AA & VA-M-EA) - As described previously [15], PTSD was diagnosed using the Structured Clinical Interview for DSM-IV Disorders (SCID) administered by trained interviewers. In accordance with the DSM-IV, PTSD consists of three symptom clusters. These include re-experiencing symptoms (B symptoms), avoidance and numbing symptoms (C symptoms) and hyperarousal symptoms (C symptoms). Total PTSD symptoms and symptom clusters (B, C, or D) were measured using the Davidson Trauma Scale for all veterans including individuals with current PTSD diagnosis and controls. The research was reviewed and approved by the Institutional Review Boards at the Salisbury VA, Hampton VA, Durham VA and Duke University Medical Centers.

Boston VA National Center for PTSD (VA-NCPTSD) – As described by Logue and colleagues [16], VA study participants were administered the CAPS, a 30-item structured diagnostic interview that assesses the frequency and severity of the 17 DSM-IV PTSD symptoms, 5 associated features and functional impairment, to assess current and lifetime PTSD symptoms. The Institutional Review Boards at two VA health care facilities approved the study.

Supplementary Figures

Supplementary Figure 1. Forest Plots of association between PTSD and *AHRR* CpGs in each cohort. N = 1,896 samples are examined. The x-axis is the effect size for each association and includes the 95% confidence interval. Error bars represent the 95% confidence interval, and the center of the error bars represent effect sizes for the CpGs in each cohort. The vertical line indicates an effect size of 0.

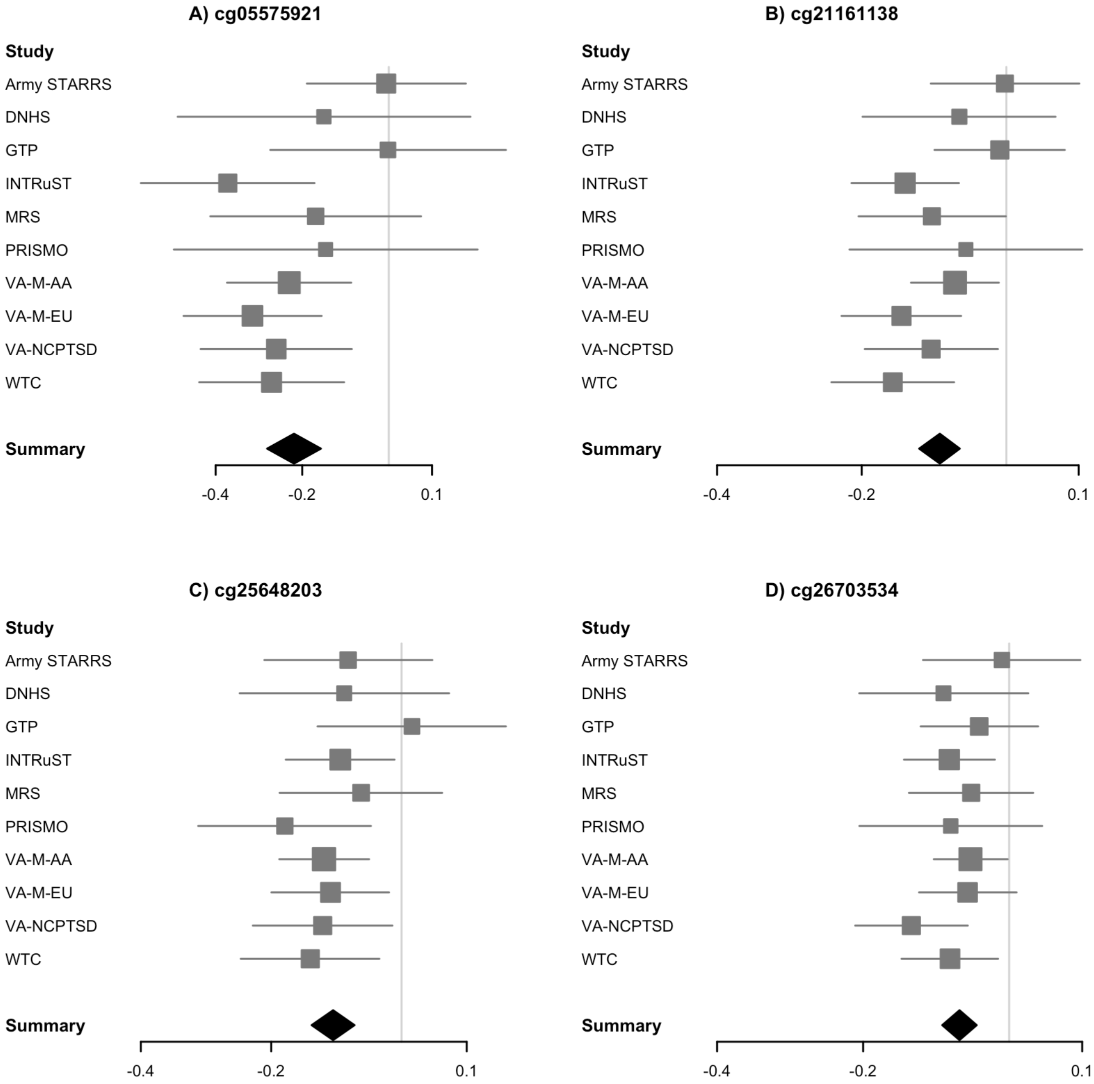

Supplementary Figure 2. Forest Plots of association between PTSD and *AHRR* CpGs stratified by sex. N = 1,896 samples are examined, including 1,303 males and 593 females. The x-axis is the effect size for each association and includes the 95% confidence interval. The y-axis representes each respective cohort. Error bars represent the 95% confidence interval, and the center of the error bars represent effect sizes for the CpGs in each cohort.

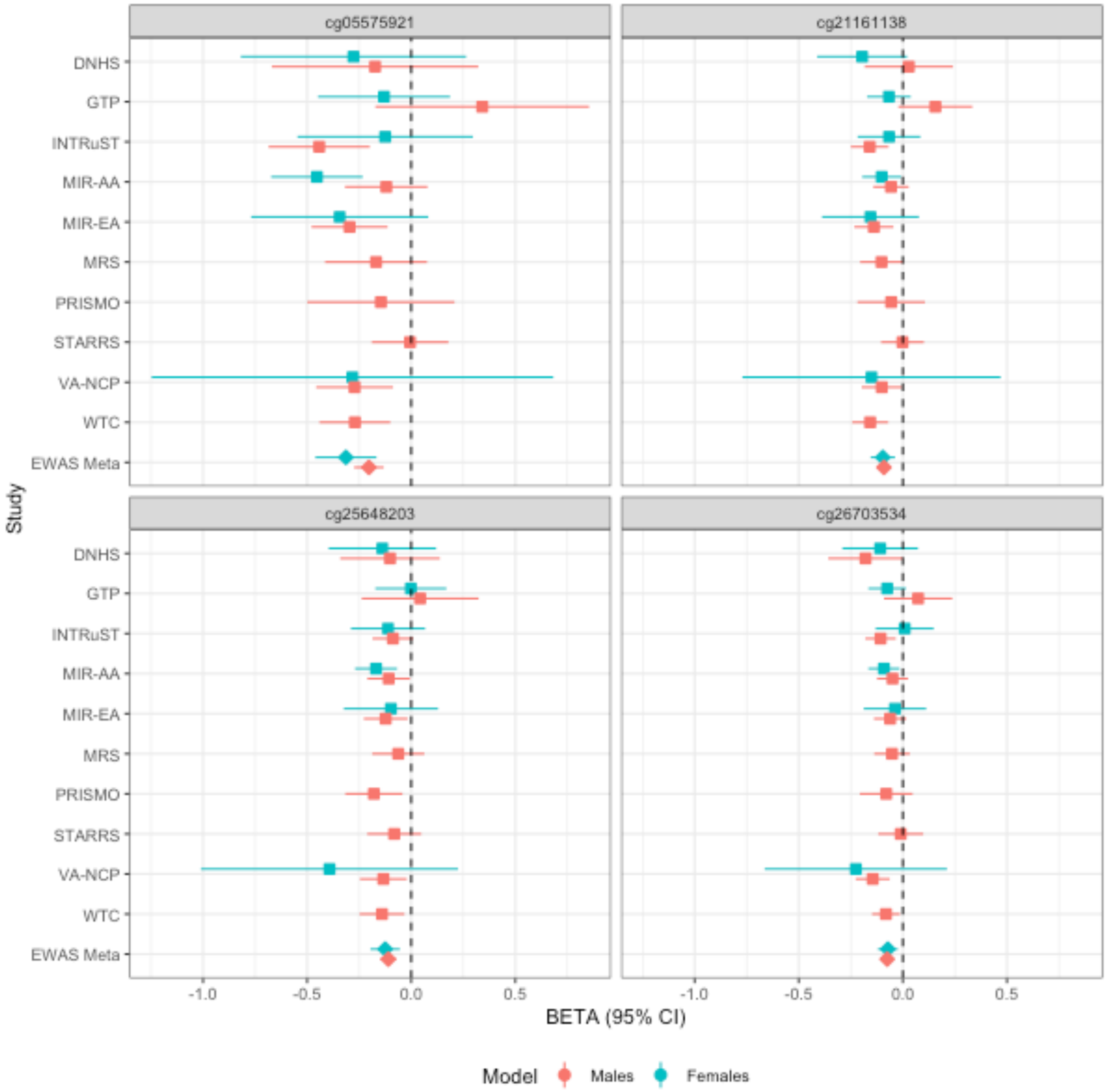

Supplementary Figure 3. Forest Plots of association between PTSD and *AHRR* CpGs stratified by European and African ancestry. N = 1,659 samples are examined, including 898 European and 761 African ancestry subjects. The x-axis is the effect size for each association and includes the 95% confidence interval. The y-axis representes each respective cohort. Error bars represent the 95% confidence interval, and the center of the error bars represent effect sizes for the CpGs in each cohort.

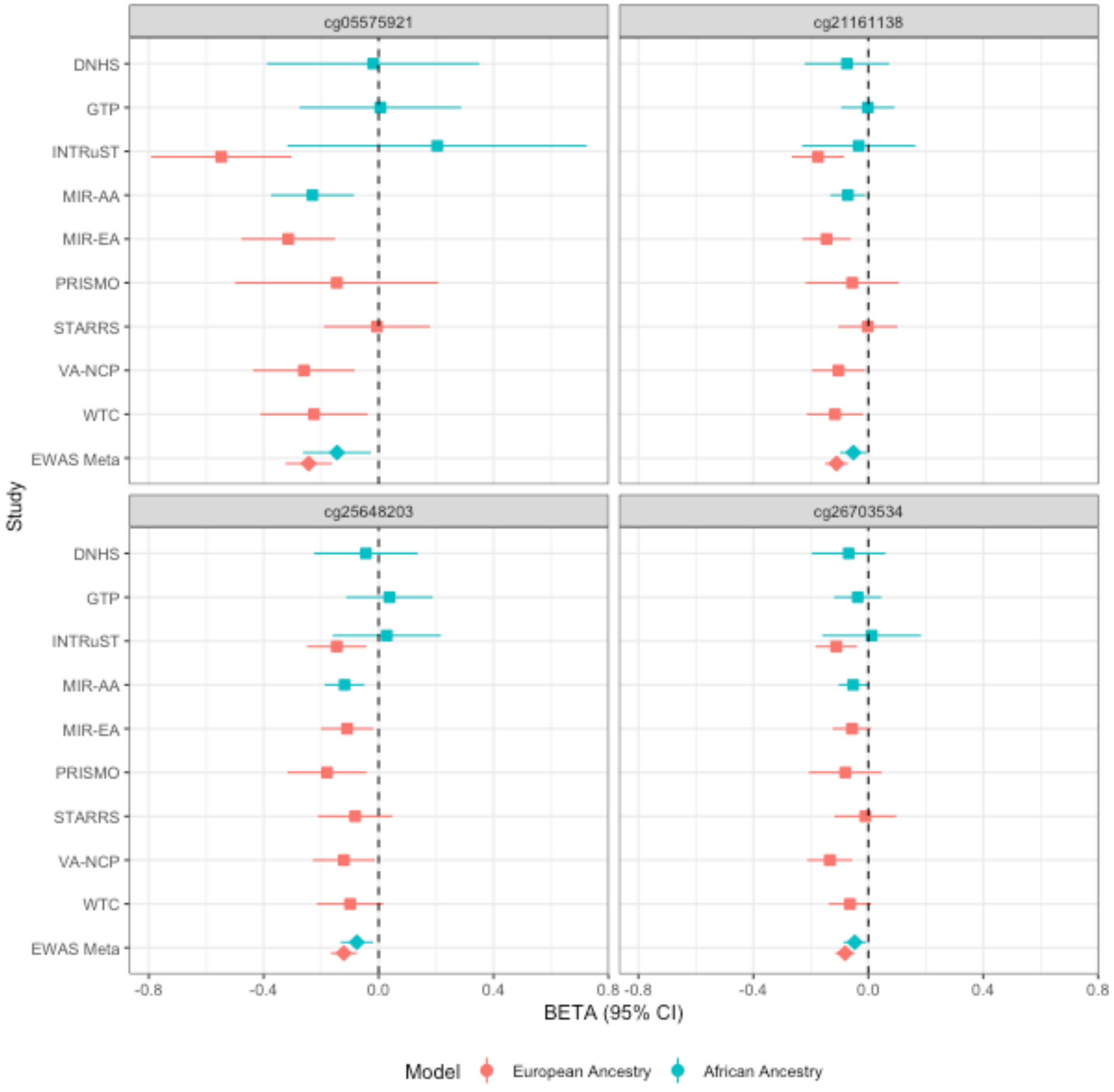

Supplementary Figure 4. Comparison of effect sizes before and after controlling for smoking. N = 1,896 samples are examined. On the x-axis, error bars represent the 95% confidence interval, and the center of the error bars represent effect sizes for each of the CpGs on the y-axis.

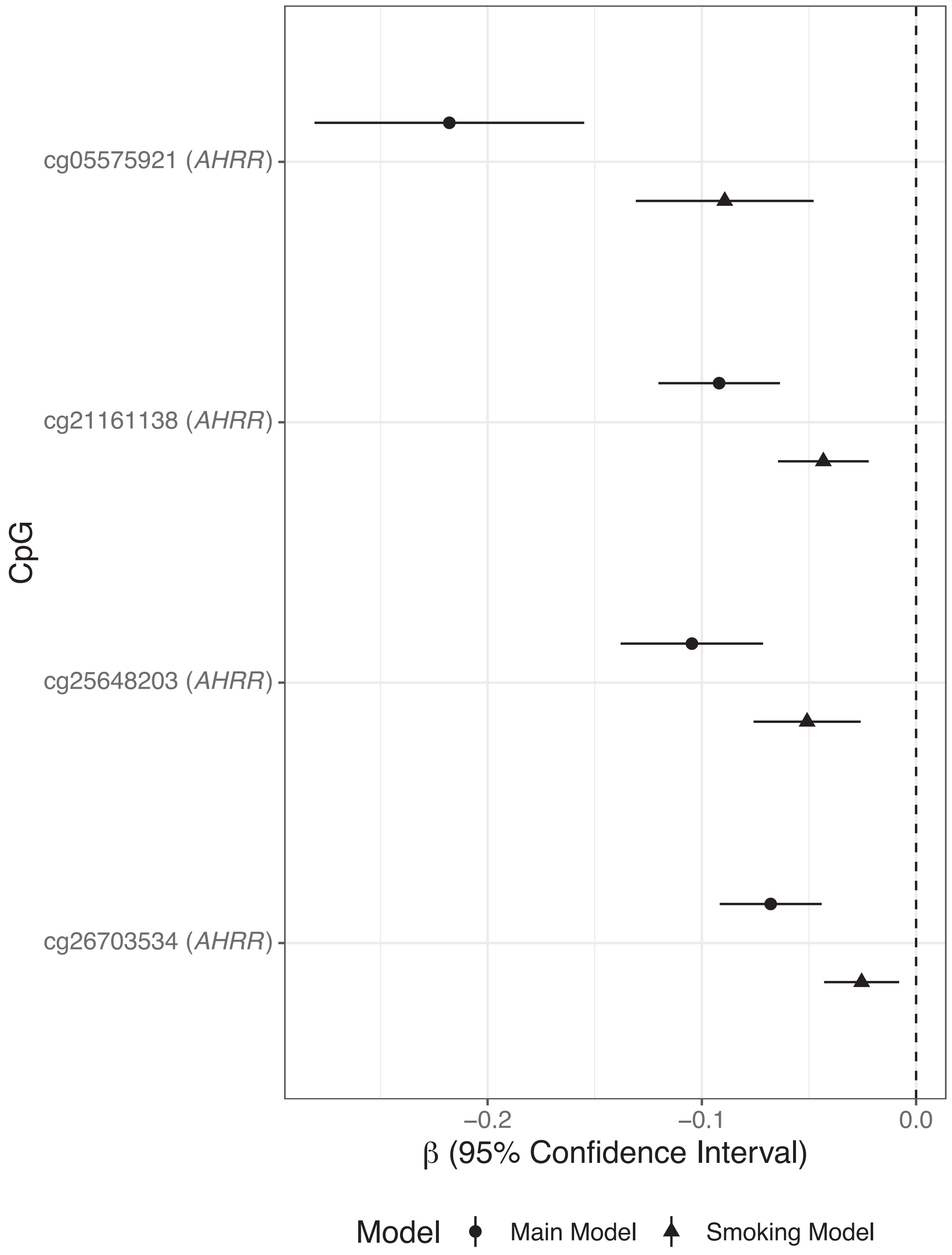

Supplementary Figure 5. Forest Plots of association between PTSD and *AHRR* CpGs stratified by smokers vs nonsmokers. N = 1,844 samples are examined, including 1,255 non-smokers (green) and 589 smokers (red). The x-axis is the effect size for each association and includes the 95% confidence interval. The y-axis representes each respective cohort. Error bars represent the 95% confidence interval, and the center of the error bars represent effect sizes for the CpGs in each cohort.

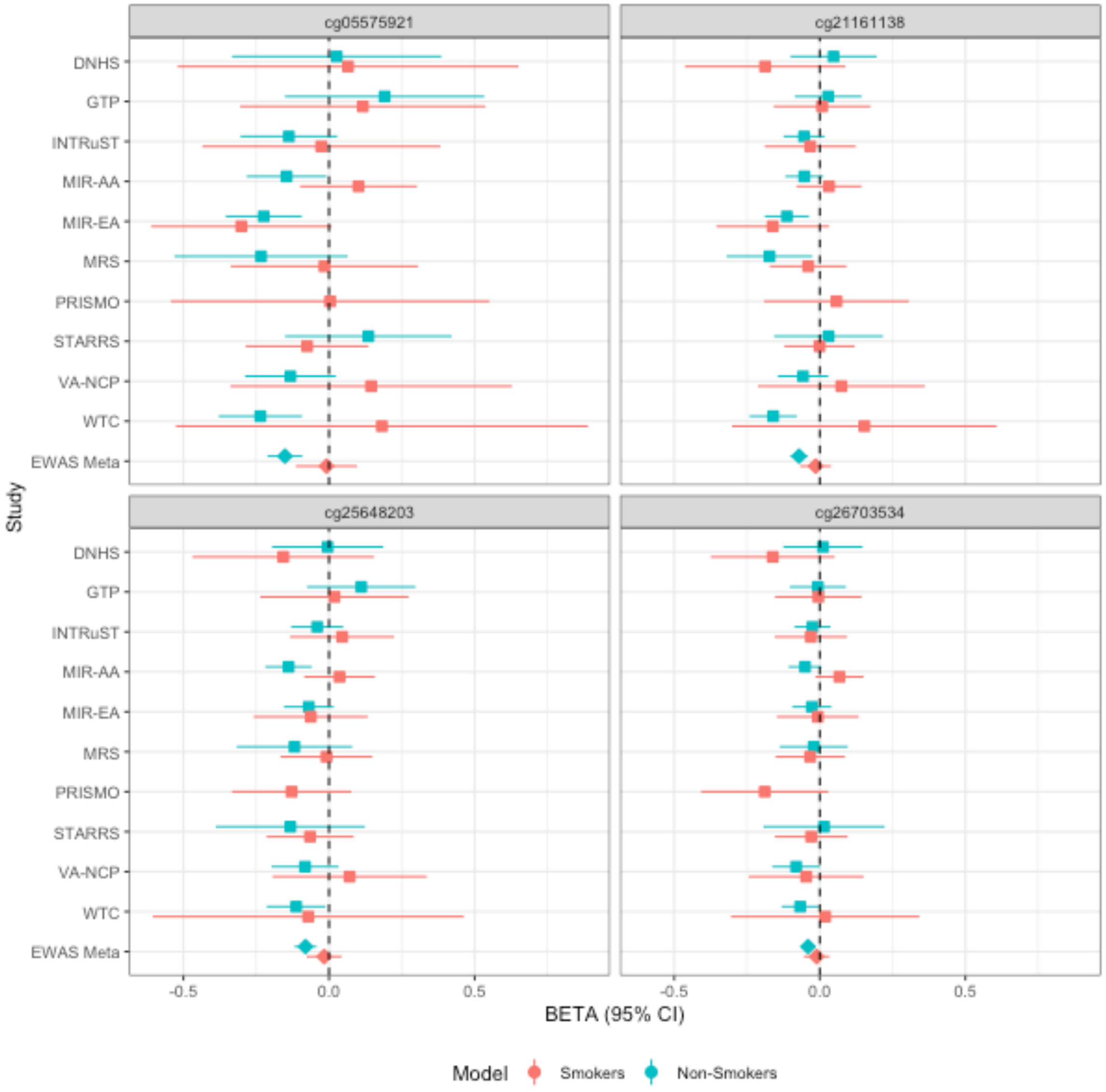

Supplementary Figure 6. Association of Smoking-Associated CpG sites with current PTSD in analyses stratified by self-reported smoking status. N = 1,844 samples are examined, including 1,255 non-smokers (circles) and 589 smokers (triangles). The x-axis is the effect size for each association and includes the 95% confidence interval. Error bars represent the 95% confidence interval, and the center of the error bars represent effect sizes for the CpGs.

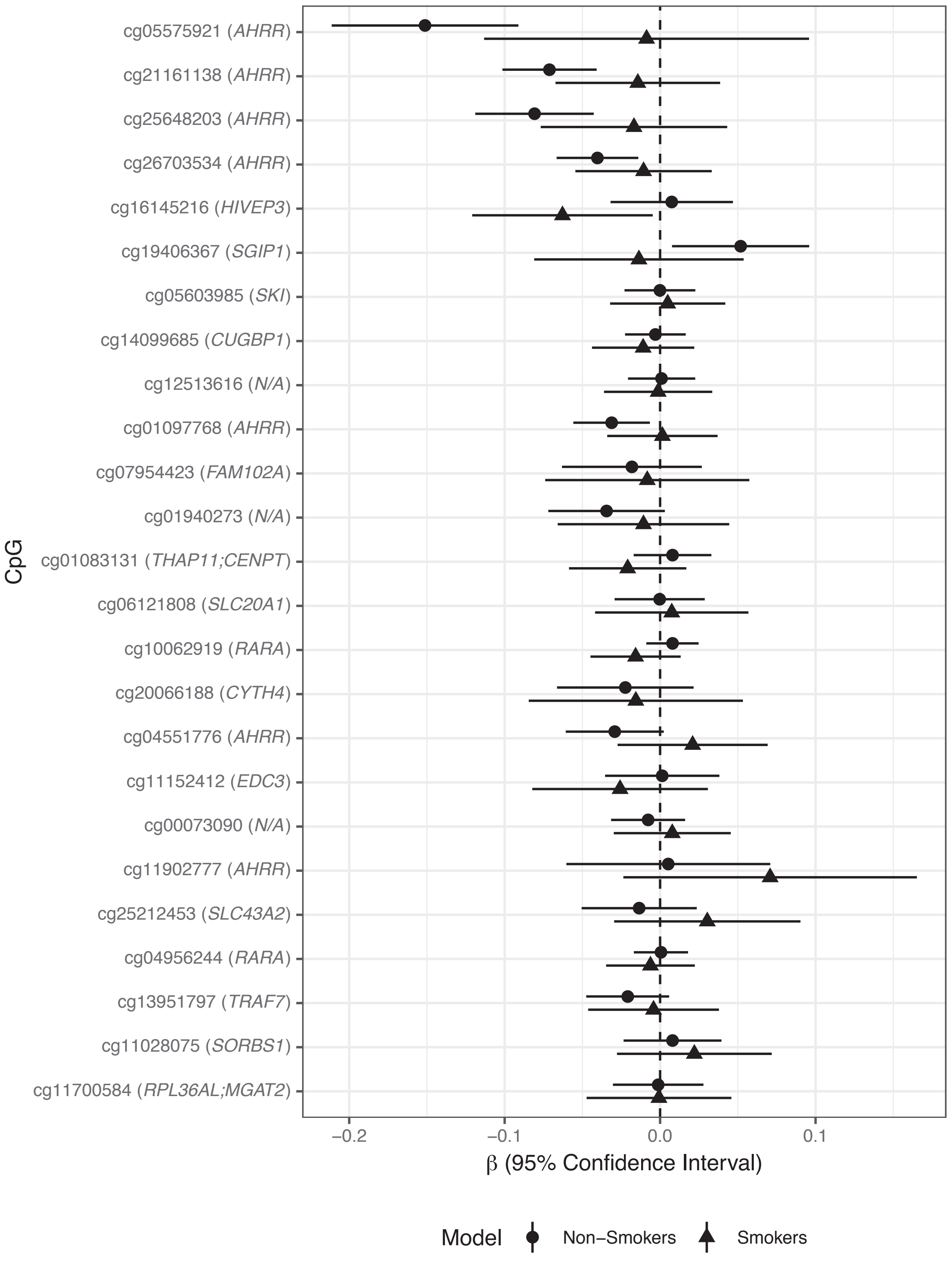

Supplementary Figure 7. Lower Kynurenine levels among PTSD cases in the MRS cohort. N = 116 samples are examined, including 53 PTSD cases and 63 controls. Box plots show median (horizontal lines), first to third quartile (box), the most extreme values within 1.5 times the interquartile range (vertical lines) and outliers (dots).

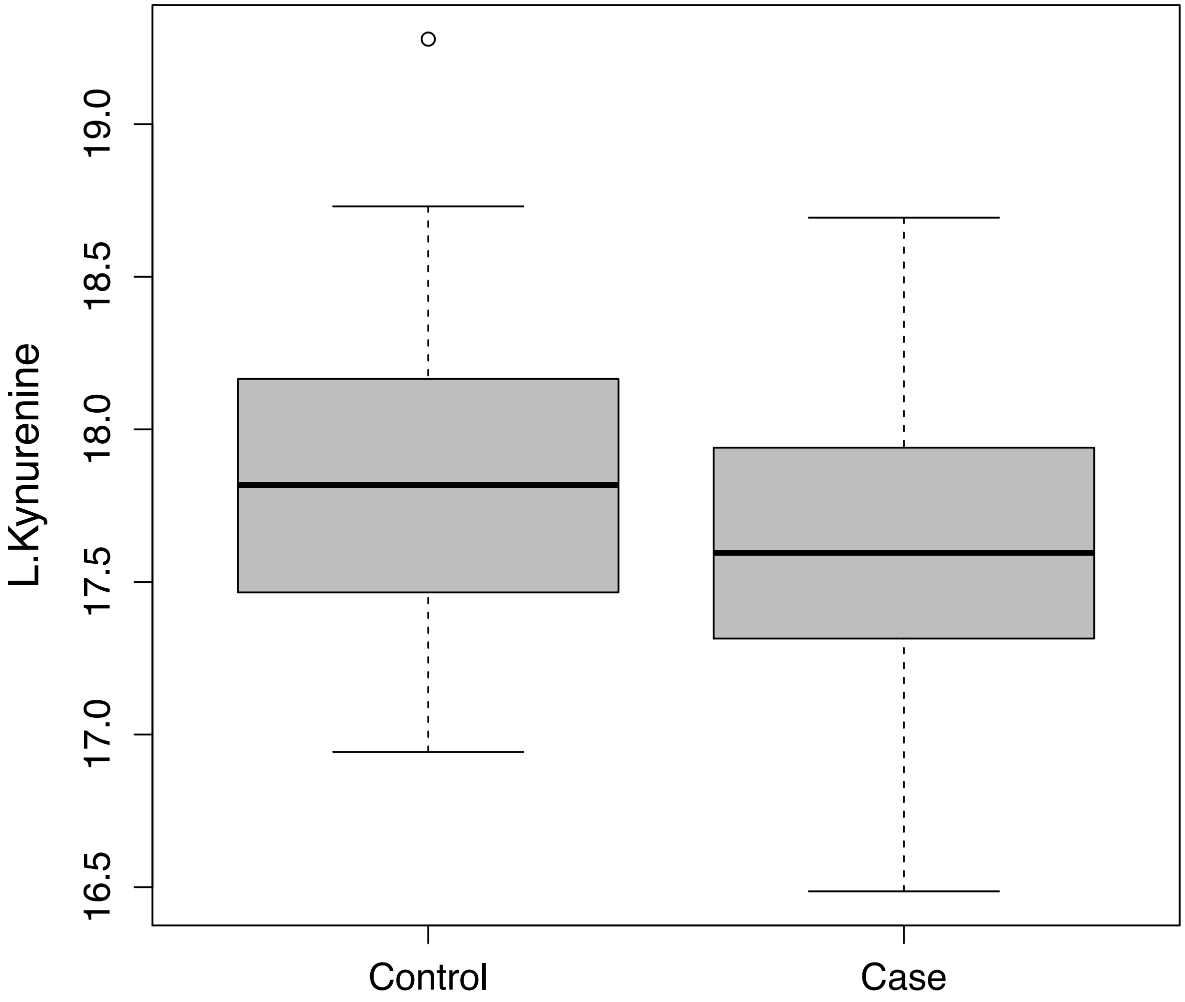

Supplementary Figure 8. Correlation of cotinine and kynurenine across all MRS subjects. Circles indicate smokers while triangles indicate non-smokers based on self-report. Correlation between cotinine and kynurenine was measured using Pearson's correlation. The Pearson correlation coefficient (r) and its corresponding nominal (unadjusted) two-sided p-value is displayed on the figure.

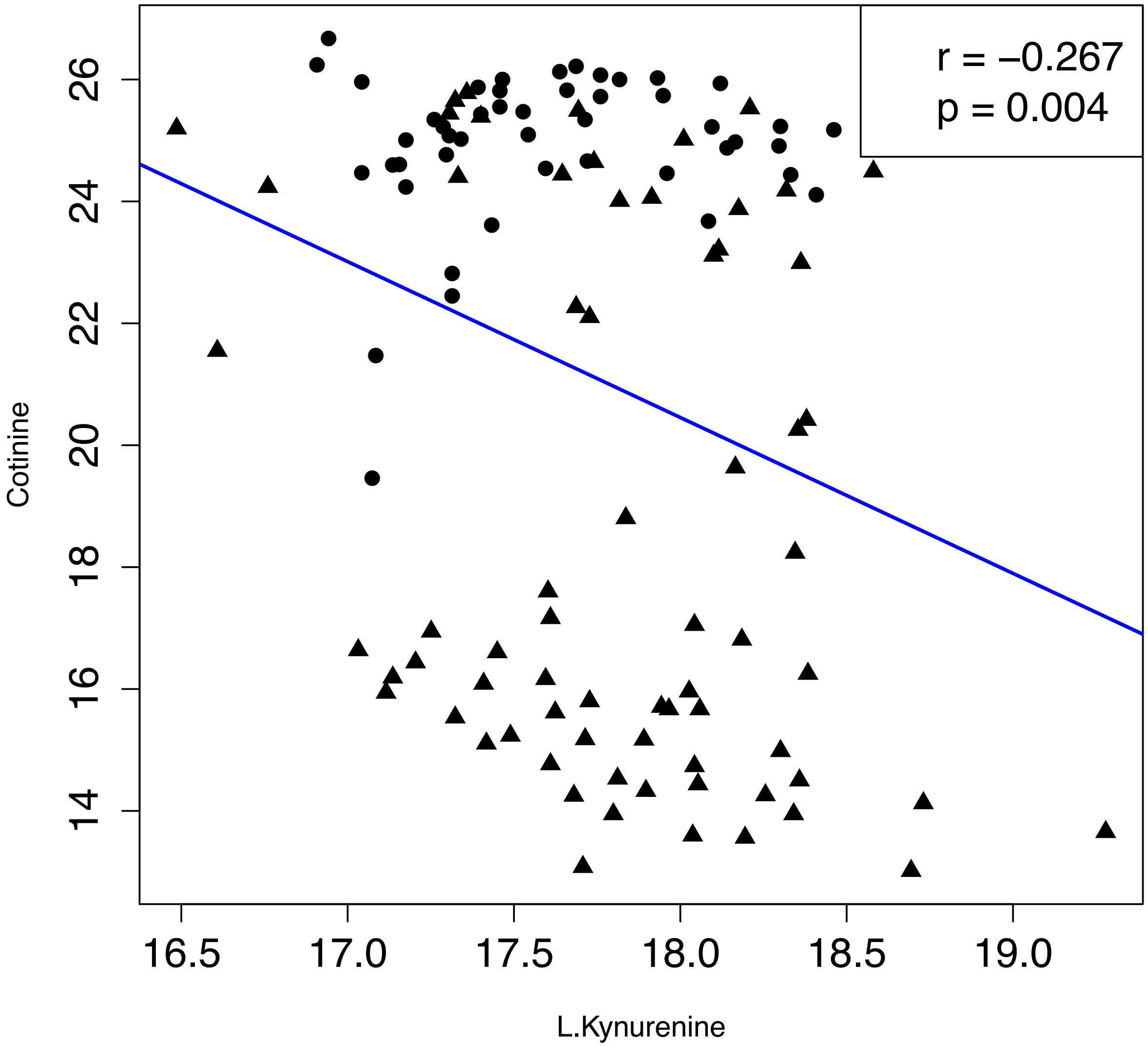

Supplementary References

1. Uddin M, Aiello AE, Wildman DE, Koenen KC, Pawelec G, de Los Santos R *et al.* Epigenetic and immune function profiles associated with posttraumatic stress disorder. *Proc Natl Acad Sci U S A* 2010; **107**(20)**:** 9470-9475.

2. Blanchard EB, Jones-Alexander J, Buckley TC, Forneris CA. Psychometric properties of the PTSD Checklist (PCL). *Behav Res Ther* 1996; **34**(8)**:** 669-673.

3. Gillespie CF, Bradley B, Mercer K, Smith AK, Conneely K, Gapen M *et al.* Trauma exposure and stress-related disorders in inner city primary care patients. *General Hospital Psychiatry* 2009; **31**(6)**:** 505-514.

4. Weathers FW, Marx BP, Friedman MJ, Schnurr PP. Posttraumatic Stress Disorder in DSM-5: New Criteria, New Measures, and Implications for Assessment. *Psychological Injury and Law* 2014; **7**(2)**:** 93-107.

5. Lecrubier Y, Sheehan DV, Weiller E, Amorim P, Bonora I, Harnett Sheehan K *et al.* The Mini International Neuropsychiatric Interview (MINI). A short diagnostic structured interview: reliability and validity according to the CIDI. *European Psychiatry* 1997; **12**(5)**:** 224-231.

6. Sheehan DV, Lecrubier Y, Harnett Sheehan K, Janavs J, Weiller E, Keskiner A *et al.* The validity of the Mini International Neuropsychiatric Interview (MINI) according to the SCID-P and its reliability. *European Psychiatry* 1997; **12**(5)**:** 232-241.

7. Herbert R, Moline J, Skloot G, Metzger K, Baron S, Luft B *et al.* The World Trade Center disaster and the health of workers: five-year assessment of a unique medical screening program. *Environ Health Perspect* 2006; **114**(12)**:** 1853-1858.

8. Bromet EJ, Hobbs MJ, Clouston SA, Gonzalez A, Kotov R, Luft BJ. DSM-IV post-traumatic stress disorder among World Trade Center responders 11-13 years after the disaster of 11 September 2001 (9/11). *Psychol Med* 2016; **46**(4)**:** 771-783.

9. Kuan PF, Waszczuk MA, Kotov R, Marsit CJ, Guffanti G, Gonzalez A *et al.* An epigenome-wide DNA methylation study of PTSD and depression in World Trade Center responders. *Translational psychiatry* 2017; **7**(6)**:** e1158.

10. Stein MB, Chen CY, Ursano RJ, Cai T, Gelernter J, Heeringa SG *et al.* Genome-wide Association Studies of Posttraumatic Stress Disorder in 2 Cohorts of US Army Soldiers. *JAMA psychiatry* 2016; **73**(7)**:** 695-704.

11. Nievergelt CM, Maihofer AX, Mustapic M, Yurgil KA, Schork NJ, Miller MW *et al.* Genomic predictors of combat stress vulnerability and resilience in U.S. Marines: A genome-wide association study across multiple ancestries implicates PRTFDC1 as a potential PTSD gene. *Psychoneuroendocrinology* 2015; **51:** 459-471.

12. Baker DG, Nash WP, Litz BT, Geyer MA, Risbrough VB, Nievergelt CM *et al.* Predictors of risk and resilience for posttraumatic stress disorder among ground combat Marines: methods of the Marine Resiliency Study. *Prev Chronic Dis* 2012; **9:** E97.

13. Boks MP, van Mierlo HC, Rutten BP, Radstake TR, De Witte L, Geuze E *et al.* Longitudinal changes of telomere length and epigenetic age related to traumatic stress and post-traumatic stress disorder. *Psychoneuroendocrinology* 2015; **51:** 506-512.

14. van Zuiden M, Geuze E, Willemen HL, Vermetten E, Maas M, Heijnen CJ *et al.* Pre-existing high glucocorticoid receptor number predicting development of posttraumatic stress symptoms after military deployment. *Am J Psychiatry* 2011; **168**(1)**:** 89-96.

15. Ashley-Koch AE, Garrett ME, Gibson J, Liu Y, Dennis MF, Kimbrel NA *et al.* Genome-wide association study of posttraumatic stress disorder in a cohort of Iraq-Afghanistan era veterans. *J Affect Disord* 2015; **184:** 225-234.

16. Logue, Baldwin, Guffanti, Melista, Wolf, Reardon *et al.* A genome-wide association study of post-traumatic stress disorder identifies the retinoid-related orphan receptor alpha (RORA) gene as a significant risk locus. *Molecular psychiatry* 2013; **18**(8)**:** 937-942.
