# Supplementary material for: Epigenome-wide meta-analysis of PTSD across 10 military and civilian cohorts identifies methylation changes in AHRR
Source: Nat Commun. 2020 Nov 24;11:5965. doi: 10.1038/s41467-020-19615-x (PMC7686485; doi:10.1038/s41467-020-19615-x)
Supplement: Supplementary file 3 — Reporting Summary [file 41467_2020_19615_MOESM3_ESM.pdf]

nature research

Corresponding author(s): Alicia K Smith

Last updated by author(s): Sep 11, 2020

Reporting Summary

Nature Research wishes to improve the reproducibility of the work that we publish. This form provides structure for consistency and transparency in reporting. For further information on Nature Research policies, see our Editorial Policies and the Editorial Policy Checklist.

Statistics

For all statistical analyses, confirm that the following items are present in the figure legend, table legend, main text, or Methods section.

n/a	Confirmed	
☐	☒	The exact sample size (*n*) for each experimental group/condition, given as a discrete number and unit of measurement
☐	☒	A statement on whether measurements were taken from distinct samples or whether the same sample was measured repeatedly
☐	☒	The statistical test(s) used AND whether they are one- or two-sided<br>*Only common tests should be described solely by name; describe more complex techniques in the Methods section.*
☐	☒	A description of all covariates tested
☐	☒	A description of any assumptions or corrections, such as tests of normality and adjustment for multiple comparisons
☐	☒	A full description of the statistical parameters including central tendency (e.g. means) or other basic estimates (e.g. regression coefficient) AND variation (e.g. standard deviation) or associated estimates of uncertainty (e.g. confidence intervals)
☐	☒	For null hypothesis testing, the test statistic (e.g. *F*, *t*, *r*) with confidence intervals, effect sizes, degrees of freedom and *P* value noted<br>*Give P values as exact values whenever suitable.*
☒	☐	For Bayesian analysis, information on the choice of priors and Markov chain Monte Carlo settings
☒	☐	For hierarchical and complex designs, identification of the appropriate level for tests and full reporting of outcomes
☐	☒	Estimates of effect sizes (e.g. Cohen's *d*, Pearson's *r*), indicating how they were calculated

Our web collection on statistics for biologists contains articles on many of the points above.

Software and code

Policy information about availability of computer code

Data collection	R v3.6.3.
Data analysis	R v3.6.3., R packages: wateRmelon v1.32, sva v3.36, limma v3.44. iMETHYL database (http://imethyl.iwate-megabank.org/)

For manuscripts utilizing custom algorithms or software that are central to the research but not yet described in published literature, software must be made available to editors and reviewers. We strongly encourage code deposition in a community repository (e.g. GitHub). See the Nature Research guidelines for submitting code & software for further information.

Data

Policy information about availability of data

All manuscripts must include a data availability statement. This statement should provide the following information, where applicable:

- Accession codes, unique identifiers, or web links for publicly available datasets
- A list of figures that have associated raw data
- A description of any restrictions on data availability

The main summary statistics data that support the findings of this study are available within Supplementary Data. The raw data for DNHS and GTP cohorts are available in the Gene Expression Omnibus database with the accession codes GSE21282 and GSE72680 respectively. Raw data for the remaining datasets are not publicly available due to them containing information that could compromise research participant privacy or consent.

April 2020

Field-specific reporting

Please select the one below that is the best fit for your research. If you are not sure, read the appropriate sections before making your selection.

- [x] Life sciences
- [ ] Behavioural & social sciences
- [ ] Ecological, evolutionary & environmental sciences

For a reference copy of the document with all sections, see nature.com/documents/nr-reporting-summary-flat.pdf

Life sciences study design

All studies must disclose on these points even when the disclosure is negative.

Sample size	No a priori sample size was selected for this meta-analysis. All cohorts and samples that met the inclusion criteria (described in Methods) were used.
Data exclusions	As part of methylation QC, Samples with probe detection call rates <90% and those with an average intensity value of either <50% of the experiment-wide sample mean or <2,000 arbitrary units (AU) were excluded. Probes with detection p-values >0.001 or those based on less than three beads were set to missing as were probes that cross-hybridized between autosomes and sex chromosomes 50. CpG sites with missing data for >10% of samples within cohorts were excluded from analysis.
Replication	We did not proceed to the replication of our findings to increase the power of discovery by meta-analyzing all 10 contributing cohorts.
Randomization	No participants were randomized in this study as we analyze observational data in our study.
Blinding	Blinding was not relevant to our study as there were not experiments done separately on case/control groups.

Reporting for specific materials, systems and methods

We require information from authors about some types of materials, experimental systems and methods used in many studies. Here, indicate whether each material, system or method listed is relevant to your study. If you are not sure if a list item applies to your research, read the appropriate section before selecting a response.

Materials & experimental systems

n/a	Involved in the study	
x		Antibodies
x		Eukaryotic cell lines
x		Palaeontology and archaeology
x		Animals and other organisms
	x	Human research participants
x		Clinical data
x		Dual use research of concern

Methods

n/a	Involved in the study	
x		ChIP-seq
x		Flow cytometry
x		MRI-based neuroimaging

Human research participants

Policy information about studies involving human research participants

Population characteristics	This study includes 878 PTSD cases and 1018 controls from 3 civilian and 7 military cohorts. Of the 1896 total participants, 55% are male, 31% are smokers, 52% are White, 41% are Black, 3% are Hispanic and 3% are unknown or from other ancestries.
Recruitment	Studies that contributed data to this manuscript are case-control studies primarily based either in US or Europe. In total 10 studies provided core data on disease status, age, sex, self-reported smoking status.
Ethics oversight	DNHS was approved by the institutional review board at the University of Michigan and University of North Carolina at Chapel Hill. GTP was approved by the Institutional Review Boards of Emory University School of Medicine and the Research Oversight Committee of Grady Memorial Hospital. WTC was approved by the Committees on Research Involving Human Subjects at Stony Brook University. Army Starrs was approved by approved by the Human Subjects Committees of the Uniformed Services University of the Health Sciences for the Henry M. Jackson Foundation (the primary grantee), the Institute for Social Research at the University of Michigan (the organization collecting the data), and all other collaborating organizations. INTRuST was approved by the Human Research Protection Program (HRPP) at UCSD, as by all the IRBs at participating sites. MRS was approved the University of California – San Diego Institutional Review Board. PRISMO was approved by the Institutional Review Board of the University Medical Center Utrecht (Utrecht, the Netherlands). VA-M-AA & VA-M-EA were approved by the Institutional Review Boards at the Salisbury VA, Hampton VA, Durham VA and Duke University Medical Centers. VA-NCPTSD was approved by Institutional Review Boards at two VA health care facilities.

Note that full information on the approval of the study protocol must also be provided in the manuscript.
