# Supplementary material for: Epigenome-wide meta-analysis of PTSD across 10 military and civilian cohorts identifies methylation changes in AHRR
Source: Nat Commun. 2020 Nov 24;11:5965. doi: 10.1038/s41467-020-19615-x (PMC7686485; doi:10.1038/s41467-020-19615-x)
Supplement: Supplementary file 4 — Description of Additional Supplementary Files [file 41467_2020_19615_MOESM4_ESM.pdf]

Description of Additional Supplementary Files

Supplementary Data 1
A table detailing clinical and demographic phenotypes by cohort

Supplementary Data 2
The complete results for all CpGs included in the meta-analysis
